# Conditional indirect genetic effects of caregivers on brood in the clonal raider ant

**DOI:** 10.1093/beheco/arad033

**Published:** 2023-04-28

**Authors:** Patrick K Piekarski, Stephany Valdés-Rodríguez, Daniel J C Kronauer

**Affiliations:** Laboratory of Social Evolution and Behavior, The Rockefeller University, New York, NY 10065, USA; Laboratory of Social Evolution and Behavior, The Rockefeller University, New York, NY 10065, USA; Howard Hughes Medical Institute, New York, NY 10065, USA; Laboratory of Social Evolution and Behavior, The Rockefeller University, New York, NY 10065, USA; Howard Hughes Medical Institute, New York, NY 10065, USA

**Keywords:** behavior, gene–environment interactions, maternal effects, parental care, parent–offspring interactions, phenotypic plasticity

## Abstract

Caregivers shape the rearing environment of their young. Consequently, offspring traits are influenced by the genes of their caregivers *via* indirect genetic effects (IGEs). However, the extent to which IGEs are modulated by environmental factors, other than the genotype of social partners (i.e., intergenomic epistasis), remains an open question. Here we investigate how brood are influenced by the genotype of their caregivers in the clonal raider ant, *Ooceraea biroi*, a species in which the genotype, age and number of both caregivers and brood can be experimentally controlled. First, we used four clonal lines to establish colonies that differed only in the genotype of caregivers and measured effects on foraging activity, as well as IGEs on brood phenotypes. In a second experiment, we tested whether these IGEs are conditional on the age and number of caregivers. We found that caregiver genotype affected the feeding and foraging activity of colonies, and influenced the rate of development, survival, body size, and caste fate of brood. Caregiver genotype interacted with other factors to influence the rate of development and survival of brood, demonstrating that IGEs can be conditional. Thus, we provide an empirical example of phenotypes being influenced by IGE-by-environment interactions beyond intergenomic epistasis, highlighting that IGEs of caregivers/parents are alterable by factors other than their brood’s/offspring’s genotype.

## Teaser

Genes of caregivers indirectly influence the growth and development of young, but how these effects may be altered by the environment is unknown. Here, we find that differences between brood reared by different genetic lineages of caregivers depend on the physiological and environmental context, namely the age and number of caregivers. Thus, how offspring traits are shaped by their caregivers’ genes is modulated by additional factors.

## INTRODUCTION

In social species, the phenotypes of individuals are influenced by the genotypes of their social partners through indirect genetic effects (IGEs), where genes expressed in one individual influence the phenotype of another individual by shaping the environment experienced by that individual. A classic example of IGEs is found in mammalian maternal care, where the genotype of the mother influences maternal performance, and consequently offspring growth and survival (Peripato and Cheverud 2002). Since the social environment of a focal individual is influenced by the genotype of its social partners, traits influenced by IGEs have a heritable environmental component (Moore et al. 1997; Wolf 2003). Thus, IGEs can influence evolutionary responses to selection, resulting in either a slowing or acceleration of phenotypic evolution (Moore et al. 1997; Wolf et al. 1998; McGlothlin et al. 2010). A complete picture of how any trait evolves requires knowledge of the extent to which it is shaped by both direct genetic effects (DGEs) and IGEs.

The relationship between a trait’s expression and the environment (i.e., the reaction norm) can differ across genotypes. Genotypes may produce the same phenotype in one environment but different phenotypes in another environment, and such conditional DGEs are indicative of genotype-specific reaction norms (i.e., gene-by-environment interactions). For example, in *Drosophila pseudoobscura*, genotypes with the fastest development at one temperature do not differ from other genotypes at other temperatures (Gupta and Lewontin 1982). If the phenomenon of gene-by-environment interactions extends to IGEs, then conditional IGEs should be ubiquitous in social species due to variability in how genotypes of social partners respond to environmental variation. Genotype-by-genotype (GxG) epistasis, wherein the magnitude and direction of a genetic effect on an individual’s phenotype depends upon the genotypes of social partners, may be considered a specific type of IGE-by-environment interaction and has been documented across a broad range of taxa for a variety of traits (Linksvayer 2007; Linksvayer et al. 2009; Buttery et al. 2010; Marie-Orleach et al. 2017; Rode et al. 2017; Culumber et al. 2018; Jaffe et al. 2020; Rebar et al. 2020; Walsh et al. 2022). However, reports of IGE-by-environment interactions beyond GxG epistasis, such as IGEs that depend on abiotic environmental factors or physiology, remain sparse (Bailey and Desjonquères 2021; Fisher et al. 2021). A rare example comes from *Drosophila*, where the direction and magnitude of IGEs that males have on female locomotion differs across male genotypes, but the difference across genotypes depends on the presence/absence of ethanol in the medium (i.e., male genotype interacts with the abiotic environment to influence female locomotion) (Signor et al. 2017). In social species, a trait’s evolution is necessarily influenced by IGEs, but also by how these IGEs change in direction and magnitude across different environments. Thus, investigations into how IGEs change across environments are needed.

Where parental care has evolved, the phenotype and fitness of offspring is heavily influenced by the genotype of their caregivers (Hunt and Simmons 2002; Mcadam et al. 2002; Peripato and Cheverud 2002; Wilson et al. 2005). Similarly, in eusocial insects where sibling workers cooperatively care for brood as allomothers, the genotypes of caregivers will have an indirect effect on the phenotype of brood by shaping the rearing environment (Linksvayer and Wade 2005). In honeybees, cross-foster experiments using low and high pollen-hoarding strains show that the larval rearing environment (i.e., whether being reared by low- or high-pollen hoarding workers) had an influence on the body mass, sucrose responsiveness and ovariole number of the resulting adults, implying IGEs (Pankiw et al. 2002; Linksvayer et al. 2009). IGEs of workers on brood have also been shown in *Temnothorax* ants, where the body mass of resulting adults is influenced by the species of worker rearing them (Linksvayer 2006, 2007). Given the strong dependence of brood on their caregivers in eusocial insects, caregiver–brood interactions offer a promising avenue to explore if and how IGEs are tuned by context.

Determining the environmental factors that shape caregiver IGEs on brood phenotypes is not straightforward in a typical eusocial insect, where age demographics, genetic heterogeneity among workers and brood, heterogeneity in sex and developmental stages of larvae, and variability in colony size confound the IGEs between caregivers and brood. All these factors can be experimentally controlled in the clonal raider ant, *Ooceraea biroi*, making this species a tractable study system to test what environmental factors modify the IGEs that caregivers have on the brood. Colonies of *O. biroi* are queen-less but contain two sub-castes of workers that differ in morphology and physiology (Ravary and Jaisson 2004). Intercastes have four to six ovarioles and tend to have vestigial eyes and a larger body size compared to regular workers, which have two ovarioles and lack vestigial eyes. *O. biroi* reproduces asexually, with all colony members being near-identical clones and (almost always) female (Kronauer et al. 2012). In the presence of larvae, workers forage and their ovaries are inactive (Ravary and Jaisson 2002; Ravary et al. 2006; Chandra et al. 2018). Coincident with the end of larval development and the onset of metamorphosis, workers stop foraging and reactivate their ovaries. Thus, colonies alternate between a reproductive phase, in which larvae are absent and adults synchronously lay eggs, and a brood care phase, in which adults tend to larvae and forage for food. This ultimately produces age-matched cohorts of brood that all eclose within a few days of each other (Ravary and Jaisson 2002, 2004), permitting experimental control over the age of both caregiving adults and brood. For example, age-matched caregivers of varying genotypes can all be supplied brood of the same age and genotype, allowing experimenters to investigate how genetic variability among caregivers influences brood growth and development (i.e., IGEs).

A previous cross-fostering experiment using two different clonal lineages (i.e., lines) showed a significant effect of caregiver genotype, brood genotype, and a GxG interaction on the proportion of brood that developed into intercastes (Teseo et al. 2014). A more recent study identified a larger range of IGEs and DGEs on both caregiver and brood phenotypes by conducting developmental, morphological, and behavioral tracking analyses in a full-factorial cross-foster experiment using four lines (Jud et al. 2022). Both caregiver and brood genotype influenced length of larval development and the proportion of time that caregivers spent in the nest, while a GxG interaction influenced the body size and intercaste proportions of brood (Jud et al. 2022). However, to date, it has not been formally tested if any of the previously reported IGEs of workers on brood are modified by environmental factors beyond GxG epistasis in any eusocial insect. More broadly, there is a paucity of studies that investigate how IGEs vary across environments, including IGEs of (allo)parents on offspring. Thus, we first tested the effects of caregiver genotype on brood phenotypes, as well as on the foraging and feeding activity of colonies. Then, in a second experiment, we experimentally manipulated the age and number of caregivers to test if caregiver IGEs are modifiable by physiological and/or environmental factors. We found that caregiver effects on brood phenotypes were altered by context, showcasing an empirical example of IGE-by-environment interactions related to (allo)parental care.

## MATERIALS AND METHODS

### Animal maintenance and genotypes used

Stock colonies were maintained in climate-controlled rooms, at 24 ± 1 °C and ≥60% relative humidity in sealed plastic containers with a plaster of Paris floor. Stocks were fed Monday, Wednesday, and Friday during the brood care phase with frozen *Solenopsis invicta* larvae and pupae. For Experiment 1, we collected recently eclosed adults (i.e., callows) from stocks C17 (line A), STC6 (line B), BG9 (line D), and BG14 (line M) to serve as caregivers, and eggs from stock STC6 (line B) to serve as the focal brood. We used young adult workers (31–35 days old) as caregivers because young worker ants primarily engage in nursing brood but can also perform tasks regularly performed by older workers (Robinson 1992; Ulrich et al. 2021). For Experiment 2, we collected recently eclosed callows from stocks C16 (line A) and STC6 (line B) to serve as caregivers, and eggs and/or first instar larvae from two different stocks of STC6 (line B) to serve as the focal brood. Stock C16 and C17 (line A) are lineages originating from Okinawa, Japan; all STC6 stocks (line B) originated from St. Croix in the U.S. Virgin Islands (Kronauer et al. 2012). BG9 (line D) and BG14 (line M) were collected in Bangladesh (Trible et al. 2020). Lines A and B are globally invasive lines nested within a clade of lines native to Bangladesh, which includes lines D and M. Phylogenetic analysis using five loci (*cytochrome oxidase I, cytochrome oxidase II, wingless, elongation factor 1*α, and *long wavelength rhodopsin*) shows that all four lines are closely related, with a reported genetic distance between any pair below 0.02 (Trible et al. 2020). Due to their close relationship, we did not expect major incompatibilities between the adults and larvae of the different lines. At the same time, previous work has shown that line A and B caregivers differ in foraging activity (Ulrich et al. 2021) and in the proportion of intercastes they rear (Teseo et al. 2014). We therefore expected that even closely related lines would show differences in phenotype.

### Experiment 1: Caregiver genotype effects on feeding, foraging and brood traits

#### Experimental design and setup

**Figure 1.**
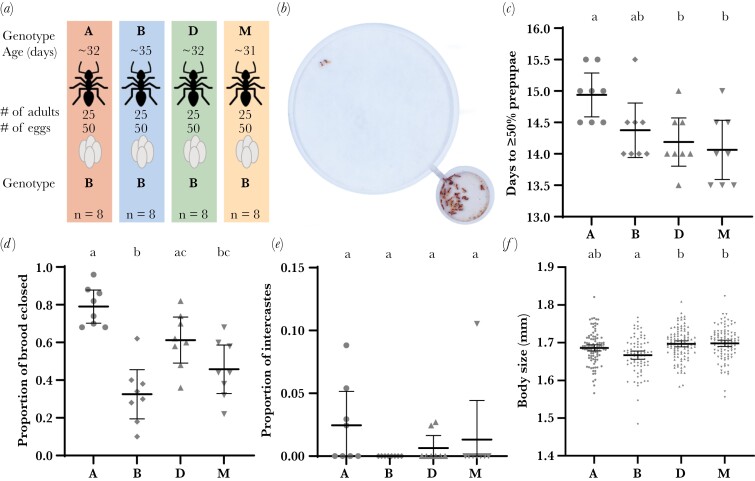
(a) Experimental design to test the effects of caregiver genotype on the growth and development of brood. (b) Raiding arenas used to house the experimental colonies, with a small nest chamber and larger foraging chamber connected by a narrow tunnel; image from (Kronauer 2020). (c) Broods’ duration of larval development, (d) survival, (e) intercaste proportions, and (f) body size in response to caregiver genotype (x-axis). For c-e, each point represents a colony. For *f*, each point represents an individual ant. Letters indicate significant differences; bars represent 95% CIs of the mean.

We generated eight experimental colonies for each of four lines (A, B, D, and M) that were similar in every aspect except for the genotype of caregivers—the reproductive physiology, age and number of caregivers, the age, number and genotype of brood, and diet were controlled (Figure 1a; Supplementary Table S1). Because we sourced all brood from a single stock colony to control for any maternal effects, variability generated in brood phenotypes between treatments must stem from genetic (or perhaps, non-genetic, environmentally induced) differences between caregivers. To minimize non-genetic differences between caregivers, we sourced them from stock colonies maintained in a climate-controlled environment and isolated them shortly after eclosion prior to the experiment (thereby partly controlling pre- and post-imaginal environmental variability).

Experimental colonies were initially composed of 30 regular callow workers (3–7 days old) in 50 mm diameter Petri dishes with a plaster of Paris floor (see Supplementary Figure S1 for a schematic of the experimental setup). Each colony was fed fire ant brood every 48 h, until eggs were laid. All colonies were maintained at 24 ± 1 °C. Each colony was transferred to a raiding arena (Figure 1b) 22 days later, at which point colonies had eggs that were two to four days from hatching. Larvae inhibit worker ovarian activity (Ravary et al. 2006; Chandra et al. 2018), so we performed the brood swap when all colonies had second to third instar larvae, ensuring that caregivers did not lay eggs when rearing the focal cohort of brood. This also provided caregivers 6 days to settle in their new housing before swapping in the focal cohort of brood. On the day we performed the brood swap, we recorded the number of larvae that had been produced by the caregivers in each colony, fed each colony three fire ant worker pupae, and adjusted all colonies to have exactly 25 regular workers. This resulted in eight experimental colonies per line, with each colony containing 50 eggs that were 10 ± 1 days old and sourced from a single line B stock colony, and 25 caregivers of a single line that were approximately the same age across all four lines (Figure 1a).

Once all colonies had ≥50% first instar larvae, we fed the colonies fire ant worker pupae daily by putting food directly into the brood chamber. For the first five feeding events, we provided colonies with six fire ant worker pupae. For days six to nine, we fed colonies with 12 fire ant worker pupae. Then, colonies were fed 18 fire ant worker pupae until ≥50% of remaining larvae had become prepupae. After the sixth day, prior to feeding, we began recording the number of uneaten fire ant pupae from the previous feeding event. For the next 8 days, we assigned a daily feeding score for each colony on a scale from zero to six, based on the number of food items eaten (e.g., score of six if at least 16 of 18 pupae were eaten, five if 13 to 15 pupae were eaten, etc.). During the entire brood care phase, we also video recorded each colony for four hours per day just prior to feeding. Video recordings were analyzed using the tracking software anTraX (Gal et al. 2020). We estimated the daily foraging activity of all colonies by quantifying the average number of ants in the foraging chamber at any time and the total distance travelled by ants in the foraging chamber (detailed given in Supplementary Material). For each colony we recorded the date that the remaining brood were ≥50% first instar larvae, ≥50% prepupae, and ≥50% eclosed as adults. We also recorded the number of brood surviving to eclosion. The proportion of intercaste brood corresponds to the proportion with vestigial eyes (Supplementary Figure S2). Individuals from the focal brood cohort were harvested 5 to 7 days after they had eclosed as adults.

To estimate body size, we measured and summed the length of the head, thorax, and first gastral segment from a lateral view (Supplementary Figure S2). We measured the body sizes of nine random caregivers from two colonies per line (18 caregivers per line), and 12–13 of the resulting focal adults without vestigial eyes from each colony. Colonies 5, 7, and 8 of line B, and colony 1 of line M had low survival, and we were only able to measure body size for one, seven, seven, and 11 individuals, respectively. Since brood that developed vestigial eyes were a priori excluded from the body size analysis, our tests for differences across caregiver genotypes is more conservative. Images were taken with a Leica Z16 APO microscope equipped with a Leica DFC450 camera using the Leica Application Suite version 4.12.0 software (Leica Microsystems, Switzerland). Lengths of body segments were measured using ImageJ (Schindelin et al. 2012).

#### Statistical analyses

We used a one-way ANOVA with post hoc Tukey’s tests to statistically test for differences in length of larval development and brood survival across conditions. We did the same to compare the average body size of the regular workers used as caregivers across conditions—all caregivers belonging to the same line came from the same stock colony, so there are no random effects due to colony. To assess whether caregiver genotype influenced colony feeding activity over the course of the brood care phase, we summed the daily feeding scores of each colony to calculate a cumulative feeding score and then performed a one-way ANOVA. We conducted a repeated measures ANOVA on both metrics of daily foraging activity after fourth root transformation to test if caregiver genotypes differ in their foraging activities for each day. To compare the cumulative foraging activity over the entire brood care phase between caregiver genotypes, the distances travelled per day were summed and then square root transformed to produce values that met the assumptions of ANOVA. The above analyses were conducted in GraphPad PRISM 8. Our body size measurements of reared brood correspond to individual ants, some of which share the same colony, and so we applied a linear mixed model (LMM) that treated caregiver genotype as a fixed effect and colony as a random effect. LMM analyses were done in R 4.1.2 (R Core Team 2022) using the package *lme4* (Bates et al. 2015). To test if caregiver genotype influenced the propensity of brood to develop as intercastes, a generalized linear mixed model (GLMM) with binomial error and logit-link was used with caregiver genotype being treated as a fixed effect, and colony identity as a random effect. This was implemented in R using the *glmer* function. Significance of model terms was assessed using the function *Anova* in the package *car* (Fox and Weisberg 2019), and *emmeans* (Lenth 2022) was used to conduct post hoc Tukey’s tests to correct for multiple comparisons. Assumptions of statistical models were validated using the function *simulateResiduals* in the package *DHARMa* (Hartig 2022).

### Experiment 2: Conditionality of caregiver genotype effects on brood traits

#### Experimental design and setup

We performed a second experiment to test whether the effects of caregiver genotype on brood phenotypes (i.e., length of larval development, survival and intercaste proportions) are dependent on other physiological or socio-environmental factors (i.e., caregiver age and colony size). First, we established 10 colonies with 50 regular worker callows (colony size = 50) and five colonies with 25 regular worker callows (colony size = 25) for both lines A and B in Petri dishes. Caregivers of lines A and B came from stocks that began the brood care phase on the exact same day. A subset of five colonies with 50 caregivers per line were given 50 first instar larvae of line B (STC6) immediately, at which point the caregivers were 5–7 days old (young A50 and young B50 condition; Supplementary Table S2). For the remaining colonies, once their larvae began hatching, we swapped in 50 first instar larvae from a line B (STC6) stock colony (old A25, old B25, old A50, old B50 conditions; Supplementary Table S2), at which point caregivers were approximately one month old. Colonies were maintained at 24 ± 1 °C and fed every 48 h. Using the same approach as in experiment 1, we recorded the length of larval development, the total number of individuals reaching eclosion, and the proportion of brood that developed as intercastes.

#### Statistical analyses

To test what factors influenced length of larval development and brood survival, we performed linear regression (package *lme4*) on a model incorporating all main effects and possible interactions in our experimental design: ~caregiver genotype + age + colony size + caregiver genotype: colony size + caregiver genotype: age. Length of larval development was reciprocal transformed (Y=1/Y) to meet the assumption of normally distributed residuals. However, the residuals showed heteroskedasticity. Thus, we estimated heteroskedasticity-consistent standard errors (i.e., Huber-White robust standard errors) using the function *vcovHC* in the package *sandwich* (Zeileis et al. 2020), applying the HC4 estimator (Cribari-Neto 2004), to make our interval estimates and hypothesis testing valid. For brood survival, the assumptions of normally distributed and homoscedastic residuals were met. To test what factors influenced the propensity of brood to develop as intercastes, a GLMM with binomial error and logit-link was used with all factors and interactions (as shown above) being treated as fixed effects, and colony identity as a random effect. This was implemented in R using the *glmer* function. Assumptions of all models were validated using the function *simulateResiduals* in the package *DHARMa* (Hartig 2022). Significance of model terms was assessed using the function *Anova* in the package *car* (Fox and Weisberg 2019), and *emmeans* (Lenth 2022) was used to conduct *post hoc* tests while correcting for multiple comparisons with the Benjamini-Hochberg method. We used the function *emmip* of package *emmeans* to create interaction plots of estimated marginal means based on the linear models.

Lastly, for each colony comprised of 25 old caregivers (i.e., old A25 and old B25), we measured the body size of five to six haphazardly selected adult individuals from the cohort they had raised. To test if caregiver genotypes influenced the final body size of their brood, we applied a LMM with caregiver genotype as the fixed effect and colony as a random effect.

## RESULTS

### Experiment 1: Caregiver genotype effects on brood traits

Caregiver genotype had a significant effect on the length of larval development (ANOVA: *F*_3,28_ = 4.94, *P* = 0.007; Figure 1c). Length of larval development differed between brood reared by line A and D caregivers, and line A and M caregivers (Tukey’s HSD: A vs. D, μ_d_ = 0.75 days, *P* = 0.024; A vs. M, μ_d_ = 0.88 days, *P* = 0.007). Length of larval development for brood reared by line A caregivers was not significantly different from those reared by line B caregivers after correcting for multiple comparisons (Tukey’s HSD: A vs. B, *P* = 0.126). Caregiver genotype also influenced the time to eclosion of brood (ANOVA: *F*_3,28_ = 14.91, *P* < 0.0001; Supplementary Figure S3). Brood reared by line A caregivers took longer to eclose compared to all other lines (Tukey’s HSD: A vs. B, μ_d_ = 1.44 days, *P* < 0.0001; A vs. D, μ_d_ = 1.44 days, *P* < 0.0001; A vs. M, μ_d_ = 1.19 days, *P* < 0.001), with no differences between line B, D, and M caregivers.

Brood survival to eclosion was influenced by caregiver genotype (ANOVA: *F*_3,28_ = 15.97, *P* < 0.0001; Figure 1d). Line A caregivers increased the survival of brood relative to lines B and M, and D caregivers improved survival relative to B caregivers (Tukey’s HSD: A vs. B, *P* < 0.0001; A vs. M, *P* < 0.001; B vs. D, *P* = 0.002). Regarding the proportion of larvae that developed into intercastes, caregiver genotype had no significant effect (GLMM: χ²(3) = 1.27, *P* = 0.7361; Figure 1e). However, very few intercastes were produced in this experiment, resulting in low statistical power. Nevertheless, there was a trend for line A caregivers to produce more intercastes than line B caregivers (Figure 1e).

Caregiver genotype influenced the average body size of brood (LMM: χ²(3) = 12.89, *P* = 0.005; Figure 1f). Brood reared by line B caregivers attained smaller adult body sizes on average compared to those reared by line D or M caregivers (Tukey’s HSD: B vs. D, *P* = 0.0245; B vs. M, *P* = 0.0160), but not line A caregivers (Tukey’s HSD: A vs. B, *P* = 0.2510). When adding the number of eclosed siblings as a fixed effect to the LMM, it had no significant effect on the body size of brood (χ²(1) = 0.766, *P* = 0.381), and neither did length of larval development (χ²(1) = 0.056, *P* = 0.813).

### Experiment 1: Body size, feeding, and foraging across caregiver genotypes

**Figure 2.**
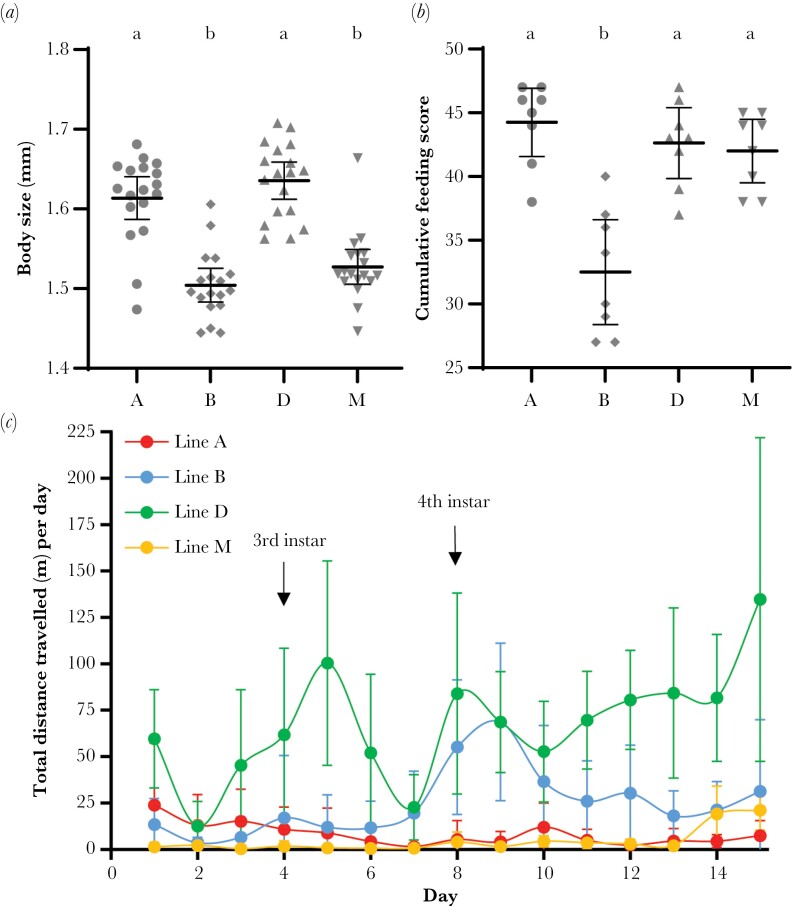
(a) Body size of caregivers used in experimental colonies across all four genotypes. (b) Cumulative feeding scores of colonies in response to caregiver genotype. (c) Total distance travelled per day by ants in the foraging chamber (a proxy for daily foraging activity of colonies) across the four caregiver genotypes. The approximate time that larvae transitioned into third and fourth instars is denoted. Letters show significant differences (a, b); bars represent 95% CIs of the mean.

Caregivers of different genotypes differed in their body size on average (ANOVA: *F*_3,68_ = 33.31, *P* < 0.0001; Figure 2a). Line A and D caregivers were larger than line B and M caregivers on average (Tukey’s HSD: A vs. B, *P* < 0.0001; A vs. M, *P* < 0.0001; B vs. D, *P* < 0.0001; D vs. M, *P* < 0.0001; A vs. D, *P* = 0.509; B vs. M, *P* = 0.469).

Caregiver genotype influenced the cumulative feeding score of colonies during the brood care phase (ANOVA: *F*_3,28_ = 16.59, *P* < 0.0001; Figure 2b). Colonies with line B caregivers ate less food relative to all other lines (Tukey’s HSD: A vs. B, *P* < 0.0001; B vs. D, *P* < 0.0001; B vs. M; *P* = 0.0001). Furthermore, a time course plot comparing daily feeding scores across the four genotypes shows that colonies with line B caregivers consumed less food than those with line A, D, and M caregivers on many days, while colonies with A, D, and M caregivers had similar feeding activity (Supplementary Figure S4a).

Caregiver genotype influenced the total distance travelled by ants in the foraging chamber during the brood care phase (ANOVA: *F*_3,28_ = 30.72, *P* < 0.0001; Supplementary Figure S4b). Caregivers of line D were most active (Tukey’s HSD: A vs. D, *P* < 0.0001; B vs. D, *P* < 0.001; D vs. M; *P* < 0.0001), and line B caregivers were more active than line A and M caregivers (Tukey’s HSD: A vs. B, *P* = 0.016; B vs. M, *P* = 0.003). Caregivers of line A and M showed no difference in the total distance travelled during the brood care phase (Tukey’s HSD: A vs. M, *P* = 0.911). When looking at patterns of daily foraging activity, both metrics for estimating foraging activity show that line D caregivers were more active than line A and M on most days, while line B caregivers were more active than lines A and M, specifically on days eight and nine, which coincides with when larvae became fourth instars (Figure 2c; Supplementary Figure S4c; Supplementary Tables S3 and S4).

In addition to differences in the average body size of regular workers (Figure 2a), we also found differences between the four lines in fecundity, reproductive maturation, and readiness to lay eggs after larvae became prepupae (Supplementary Figures S5 and S6).

### Experiment 2: Conditionality of caregiver IGEs

**Figure 3.**
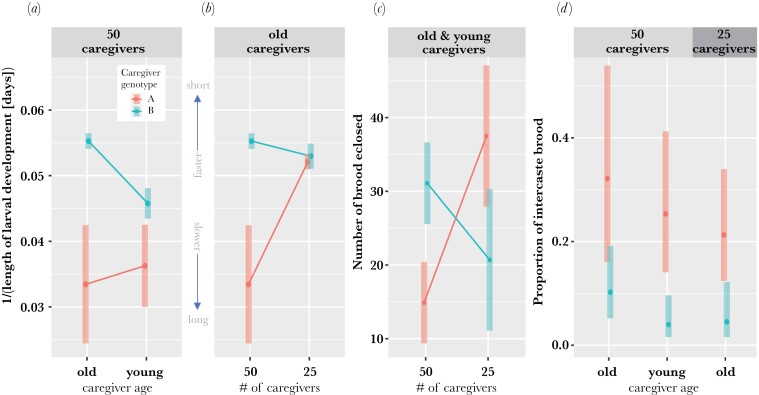
Interaction plots of the estimated marginal means for (a, b) length of larval development (reciprocal transformed), (c) brood survival, and (d) proportion of intercaste brood reared across conditions. Experimental colonies were comprised of either 25 or 50 caregivers of line A (red) or line B (blue). For colony sizes of 50 caregivers, caregivers were either ~1 month old or ~1 week old (i.e., old vs. young). For colony sizes of 25, caregivers were ~1 month old. Error bars represent 95% CIs of the mean. Violin plots are shown in Supplementary Figure S9.

Caregiver genotype (LM: *F*_1,24_ = 7.68, *P* = 0.011), age (*F*_1,24_ = 52.14, *P* = 1.843^-07^), an interaction between caregiver genotype and age (*F*_1,24_ = 5.11, *P* = 0.033), and an interaction between caregiver genotype and colony size (*F*_1,24_ = 21.44, *P* = 1.062^-04^) had a significant effect on the length of larval development. Interaction plots of the estimated marginal means (and 95% CIs) show that caregiver genotype and age interacted (Figure 3a), and caregiver genotype and colony size interacted (Figure 3b) to influence length of larval development. For colonies with 50 caregivers, larvae reared by young line B caregivers had longer development compared to those reared by old B caregivers (post hoc test: *P* < 0.0001; Supplementary Table S5), but larvae reared by young A caregivers did not differ from those reared by old A caregivers (post hoc test: *P* = 0.645; Supplementary Table S5; Figure 3a). When comparing old A caregivers at colony sizes of 25 and 50, larger colony size was associated with longer larval development (post hoc test: *P* < 0.001; Supplementary Table S5), but not when comparing old B caregivers (post hoc test: *P* = 0.060—trend in opposite direction; Supplementary Table S5; Figure 3b). Also, there was a difference in length of larval development when comparing A and B caregivers at colony sizes of 50 (both young and old), but not at colony sizes of 25 (post hoc tests: young A50 vs. young B50, *P* = 0.013; old A50 vs. old B50, *P* = 0.0001; old A25 vs. old B25, *P* = 0.497; Supplementary Table S5), meaning a caregiver genotype effect was detected only at colony sizes of 50 (Figure 3b).

Caregiver genotype (LM: *F*_1,24_ = 5.22, *P* = 0.032) and its interaction with colony size (*F*_1,24_ = 18.96, *P* = 2.143^-04^) had a significant effect on brood survival. Interaction plots of the estimated marginal means (and 95% CIs) show that caregiver genotype and colony size interacted to influence brood survival (Figure 3c). Line A caregivers had higher brood survival at colony sizes of 25 compared to 50 (post hoc test: *P* < 0.001; Supplementary Table S6), but colony size had no effect for line B caregivers (post hoc test: *P* = 0.096—trend in opposite direction; Supplementary Table S6; Figure 3c). Also, line B caregivers outperformed line A caregivers at colony sizes of 50 (post hoc test: *P* < 0.001; Supplementary Table S6), but line A caregivers outperformed line B caregivers at colony sizes of 25 (post hoc test: *P* = 0.034; Supplementary Table S6). Thus, the direction of the caregiver genotype effect depended on colony size (Figure 3c).

Only caregiver genotype had a significant effect on the proportion of brood that developed into intercastes (GLMM: χ²(1) = 25.17, *P* = 5.24^-07^). The caregiver genotype effect on intercaste proportions was not conditional on context (Figure 3d; Supplementary Figure S7). All three comparisons between A and B caregivers show that a higher proportion of brood developed into intercastes when reared by line A caregivers (post hoc tests: young A50 vs. young B50, *P* = 0.004; old A50 vs. old B50, *P* = 0.042; old A25 vs. old B25, *P* = 0.019; Supplementary Table S7). When adding the number of eclosed brood as a fixed effect to the GLMM, it had no significant effect on the proportion of intercaste brood (χ²(1) = 2.02, *P* = 0.155). Lastly, a LMM shows that brood reared by line A caregivers attained a larger body size on average compared to those reared by line B caregivers (*t* = 5.28, *P* = 0.001), even when excluding intercastes from the analysis (*t* = 4.88, *P* = 0.002; Supplementary Figure S8).

## DISCUSSION

Our results show that caregiver genotype has a significant effect on the foraging and feeding activity of colonies, and on the growth and development of brood. We recover significant and robust IGEs of caregivers on length of larval development, survival, body size, and (sub)caste development. We found that caregiver genotype interacts with other factors (age and colony size) to influence length of larval development, and that differences in brood survival between caregiver genotypes can also be context dependent. Altogether, the results show that caregivers have profound and sweeping IGEs on brood phenotypes in *O. biroi*, and that these IGEs can be conditional.

### Indirect genetic effects versus indirect environmental effects

Parental effects can be genetic, where genetic variability between parents generates variability in offspring phenotype (i.e., IGEs), or non-genetic, where variability in the phenotype of parents that was environmentally induced generates variability in offspring phenotype (i.e., indirect environmental effects—also known as transgenerational plasticity) (Mousseau and Fox 1998; Weaver et al. 2004; Danchin et al. 2011; Tariel et al. 2020). Our experimental design reduces phenotypic variability across caregiver genotypes due to different environmental backgrounds. Reproducibility of caregiver genotype effects on the same brood phenotypes across studies (Teseo et al. 2014; Jud et al. 2022), and across experiments (this study), provides good evidence that these effects have some genetic basis. Thus, these effects can be classified as IGEs.

Prenatal (i.e., before egg hatching) maternal effects on caste fate have been demonstrated in some ants. In *Pogonomyrmex* seed harvester ants, only eggs laid by queens that were exposed to cold and were at least 2 years of age have the potential to develop into future queens (Schwander et al. 2008; Libbrecht et al. 2013). Prenatal maternal effects on brood development and body size have also recently been shown in honeybees (Wei et al. 2019). The contribution of non-genetic maternal effects in determining body size has not been formally investigated in the clonal raider ant. However, prenatal maternal effects do not contribute to differences in the body size and intercaste proportions of brood between caregiver genotypes in our experiments because all eggs within an experiment were sourced from the same line B stock colony, and thus the size and quality of eggs given to all caregivers were on average equal.

There can also be postnatal (i.e., after egg hatching) maternal effects on offspring body size, in which larger caregivers provision offspring more (Hunt and Simmons 2000; Kindsvater et al. 2012; Rollinson and Rowe 2016). In *Nicrophorus* burying beetles, there is transgenerational plasticity of adult body size, such that, among genetically similar mothers that have been experimentally manipulated to be either small or large, large mothers rear their brood to larger body sizes mostly via postnatal maternal effects (Steiger 2013). In contrast, the size of workers does not affect the size of brood in bumblebees (Cnaani and Hefetz 1994). In our study, line M caregivers were smaller than line D caregivers, and of similar size to line B caregivers (Figure 2a), yet they reared brood to the same body size on average as line D caregivers and to a larger body size than line B caregivers (Figure 1f). This suggests that the body size differences of caregivers across genotypes, whether genetically based or not, do not explain the differences in the average body size of brood they reared.

### Possible mechanisms of caregiver effects on brood size and caste

Our results demonstrate that caregiver genotype influences the body size and caste fate of brood in *O. biroi* through postnatal effects, but the mechanistic basis of this effect remains unknown. One hypothesis is that differences in foraging activity translate to differences in larval growth. Consistent with previous studies, we find that workers of different genotypes differ in their levels of foraging activity (Ulrich et al. 2021; Jud et al. 2022). However, the differences in foraging activity between different genotypes of caregivers do not correlate with differences in the body size of brood. Line B caregivers showed higher foraging activity than lines A and M, but lower foraging activity than line D (Figure 2c; Supplementary Figure S3b), yet brood reared by line B were on average smaller than brood reared by lines D and M (Figure 1f). Another hypothesis is that differences in the permissiveness of larval cannibalism across caregiver genotypes leads to differences in the average body sizes of brood. However, adding brood survival as a fixed effect in the linear models of both experiments showed that it had no significant effect on the body size or intercaste proportions of brood. Therefore, there is no evidence that differences in levels of larval cannibalism contribute to explaining the variation in body size and intercaste proportions, consistent with observations in other studies (Lecoutey et al. 2011; Teseo et al. 2014; Jud et al. 2022).

Many behavioral differences across caregiver genotypes could generate variation in the average body size or intercaste proportions of brood in *O. biroi*. For example, caregivers influence caste fate by biting larvae in some ant species (Brian 1973; Penick and Liebig 2012). To determine if such, or other, adult-larva interactions are explanatory, high magnification video recordings of how caregivers of different genotypes interact with larvae are needed. Another possibility is that caregiver genotype influences the quantity of nutrition received by brood. We found that colonies with line B caregivers ate significantly less food compared to all other genotypes (Figure 2b) and reared brood to smaller body sizes on average (Figure 1f). Differences in feeding activity of colonies may represent a DGE on caregiver food intake, an IGE on brood food intake, or a combination of both. Since we only measured colony-level feeding activity, we are unable to discern how food was distributed amongst adults and larvae. It is possible that certain genotypes of caregivers are more voracious, perhaps due to a higher metabolic rate or satiation threshold, and the brood received the same amount of food across caregiver genotypes. Alternatively, perhaps caregivers of different genotypes exhibit differences in the production of pheromones that act on either larvae or workers to ultimately influence body size or caste ratios. In several ant species, queen pheromone prevents sexualization of brood by affecting worker rearing behavior and also elicits workers to execute sexual brood (Vargo and Fletcher 1986; Edwards 1991; Vargo and Passera 1991; Oliveira et al. 2020). Accordingly, a fertility signal analogous to the queen pheromone in other ants has been suggested to modulate intercaste proportions in *O. biroi* (Lecoutey et al. 2011). More experiments are needed to discover the mechanisms that contribute to worker regulation of body size and caste fate of brood in *O. biroi*.

### IGE-by-environment interactions on brood development and survival

Caregiver genotype had a significant effect on length of larval development in both experiments, suggesting that genotypes differ in how they interact with their brood. This finding is corroborated by a recent study, which found that both caregiver and brood genotypes influenced the length of larval development (Jud et al. 2022). A shortcoming of our study is that we varied caregiver genotype but not brood genotype, meaning that the observed effects on brood phenotypes could be caused by either the matching status between partner genotypes (i.e., B|B = match vs. A|B = mismatch) or caregiver genotype. However, larvae reared by line A caregivers develop longer than when reared by line B caregivers regardless of brood genotype (Jud et al. 2022). Thus, the brood’s length of larval development is influenced by an IGE rather than a mismatch-of-genotypes effect (i.e., a GxG interaction). Furthermore, even if the caregiver genotype effect was dependent on the genotype of the brood (i.e., a GxG interaction), this translates to an IGE that is dependent on the genotype of the social partner and may also be modifiable by additional environmental factors.

We found that length of larval development was influenced by an interaction between caregiver genotype and age, and caregiver genotype and colony size (Figure 3a,b), suggesting that the magnitude of the IGE depended on the age and number of caregivers. For example, old line A and B caregivers showed differences in their brood’s length of larval development at colony sizes of 50, but not 25, indicating the IGE is conditional on colony size (Figure 3b). Altogether, our results highlight that the rate of development of brood depends on caregiver genotype, and that this IGE is modifiable by other physiological and/or environmental factors. Although it is not surprising to find that caregiver IGEs on the length of larval development are context-dependent, to our knowledge, this is the first study to demonstrate such an IGE-by-environment interaction in a eusocial insect.

In the alpine silver ant *Formica selysi*, brood reared by workers from monogynous colonies have better survival compared to those reared by workers from polygynous colonies (Purcell and Chapuisat 2012), and the workers-to-larvae ratio affects brood survival and body size (Purcell et al. 2012). We recovered differences in brood survival between line A and B caregivers (Figure 1d), and evidence that the direction and magnitude of this IGE is context-dependent (Figure 3c). Two previous studies that cross-fostered lines A and B found no effect of caregiver genotype on brood survival (Teseo et al. 2014; Jud et al. 2022). In the most recent study (Jud et al. 2022), colonies were comprised of 8 adults and 7 larvae, and in the first study (Teseo et al. 2014), colonies were comprised of 50 adults, but the number of larvae was not standardized (ranging from ~15 to 78 larvae). We found that line B caregivers improved brood survival relative to line A caregivers in colonies of 50 adults and 50 larvae, while line A caregivers showed better performance relative to line B caregivers in colonies of 25 adults and 50 larvae in both experiments. For some unknown reason, colonies with 50 one-month-old line A caregivers and 50 larvae had strikingly poor brood survival (Figure 3c). This implies that an interaction between colony size and caregiver genotype influences brood survival, where line A caregivers perform better at smaller colony sizes or a lower workers-to-larvae ratio. Thus, the inconsistency of caregiver genotype effects on brood survival across studies could be due to each study having different colony sizes and workers-to-larvae ratios. Our results suggest that, in *O. biroi*, the effect of caregiver genotype on brood survival is conditional on additional properties of the social environment (i.e., another IGE-by-environment interaction).

### Outlook on IGE-by-environment interactions

In the presence of IGE-by-environment interactions, it is expected that the magnitude and direction of IGEs will depend on the specific environmental conditions experienced. Therefore, detection of IGEs on a trait may differ across studies. Some IGEs may be more robust and consistent across contexts, such as the effect of caregiver genotype on intercaste proportions in *O. biroi* (Teseo et al. 2014; Figure 3d). However, even for this trait, our first experiment did not detect a significant IGE (Figure 1e), suggesting some unknown factor dampened the effect size. A recent study examined the relative impact of DGEs and IGEs on brain gene expression in recently eclosed *O. biroi* adults when cross-fostering lines B and M (Kay et al. 2022). They found that 0 genes were differentially expressed in recently eclosed adults due to IGEs, but over 1000 were differentially expressed due to DGEs. Although surprising, it is important to point out that the transcriptomic data is limited to adult brain tissue one or eight days after eclosion, so the extent that caregiver IGEs influence gene expression of brood in other tissues and during pre-imaginal development is unknown. Also, IGEs on larval phenotypes (e.g., length of development) may not necessarily translate to differences in brain gene expression later in adulthood. Furthermore, the experiment was performed under a single set of experimental conditions (i.e., colony sizes of nine adults and nine larvae), and IGEs on gene expression may also be context dependent.

The magnitude and direction of IGEs are dependent on factors other than the social partner’s genotype (i.e., GxG epistasis), yet there are not many examples reported in the literature. A conditional IGE has been well documented in the red imported fire ant, *Solenopsis invicta*, where colony social organization is under strong genetic control by a supergene haplotype, referred to as the *Social b* (*Sb*) haplotype (Wang et al. 2013; Yan et al. 2020). Monogynous colonies contain only *SB/SB* queens and workers, while polygynous colonies have multiple *SB/Sb* queens and both *SB/SB* and *SB/Sb* workers. *SB/SB* workers normally kill *SB/Sb* and supernumerary queens, but when the frequency of *SB/Sb* workers in the colony surpasses a threshold of 5–10%, *SB/SB* workers tolerate multiple *SB/Sb* queens and kill *SB/SB* queens (Ross and Keller 2002). Thus, the IGE of *SB/Sb* workers on *SB/SB* worker behavior is conditional on the proportion of heterozygous workers. In other animals, IGEs contribute more to the heritable variance of growth rate in competitive (restricted feeding) relative to non-competitive (*ad libitum* feeding) contexts, suggesting IGEs may be stronger when resources are limited (in pigs: Piles et al. 2017; in shrimp: Luan et al. 2020). In flies, male IGEs on female locomotion are dependent on abiotic factors (Signor et al. 2017). A study on red squirrels found indirect effects on neighbor’s parturition dates at high population densities and not at low densities but could not conclude if these indirect effects had a genetic basis (Fisher et al. 2019). Our study provides an example of conditional IGEs relevant to interactions between caregivers and their young.

## CONCLUSIONS

The significance of IGE-by-environment interactions in shaping traits has been largely omitted in the social insect literature. Here we found that brood phenotypes are influenced by IGEs stemming from their caregivers, and that these IGEs can be altered by the physiological and/or environmental context (i.e., the number and age of caregivers). The mechanisms by which *O. biroi* caregivers regulate their brood’s rate of development, survival, body size and caste fate remain unknown. However, by identifying caregiver genotypes that produce different rearing environments that ultimately generate differences in these brood traits, we can now begin to reveal such mechanisms and their genetic basis.

Understanding the intimate coevolution of (allo)parent and offspring phenotypes requires insights into the IGEs that both have on each other at the level of physiology, behavior, and gene expression, as well as how these IGEs are modified by other environmental factors. We stress the importance of extending a gene-by-environment framework to future research on IGEs, where measurement of IGEs across various conditions may be necessary to draw inferences about how traits shaped by social interactions evolve.

## Supplementary Material

arad033_suppl_Supplementary_Material

## Data Availability

Analyses reported in this article can be reproduced using the data provided by Piekarski et al. (2023).
