# Supplementary material for: Conditional indirect genetic effects of caregivers on brood in the clonal raider ant
Source: Behav Ecol. 2023 Apr 28;34(4):642–52. doi: 10.1093/beheco/arad033 (PMC10332452; doi:10.1093/beheco/arad033)
Supplement: arad033_suppl_Supplementary_Material [file arad033_suppl_supplementary_material.docx]

**Electronic Supplementary Materials**

“Conditional indirect genetic effects of caregivers on brood in the clonal raider ant”

**Supplementary Methods**

*Foraging activity metrics*

We estimated the daily foraging activity of each colony by tracking colonies for four hours per day, starting 20 hours after the previous feeding event. In other words, colonies were fed everyday at midnight and video recorded from 8pm to midnight. We used the tracking software anTraX ([1](#_ENREF_1)) to analyze videos. Using the software, we first specified the region of interest, which only included the foraging chamber. Thus, when an ant (or other object) is detected in the foraging chamber, anTraX creates a track and outputs the spatial coordinates of that object frame-by-frame, until that track ends (which occurs when the object merges with another object or leaves the foraging chamber). Individual ants were not paint marked, so we could only estimate the global foraging activity of a colony, without knowing the contribution of each individual to that effort. From this xy coordinate output, we calculated (i) the average number of objects present in the foraging chamber at any point in time, and (ii) the total distance travelled by all objects in the foraging chamber per day.

To estimate (i), we took the length of the output file and divided it by the total number of frames recorded that day. This is effectively equivalent to dividing the sum of the number of tracks present for each individual frame by the total number of frames, giving the average number of tracks per frame (see ants_in_arena.py). To estimate (ii), we calculated the distance between each consecutive pair of coordinates that belonged to the same track, and then summed the distances for each four-hour video. This provides a measure of the total distance travelled by ants in the foraging chamber for each day (see distance_travelled.py).

We initially expected that the second metric (ii) would be a more accurate measurement of foraging activity because it excludes the contribution of dead ants in the foraging chamber and other objects incorrectly classified as ants, such as excrement. Both factors would inflate the first metric. However, both metrics were highly correlated (*R*^2^ = 0.9133), suggesting that these potential issues did not contribute substantially.

**References**

1. Gal A, Saragosti J, Kronauer DJC. 2020 anTraX, a software package for high-throughput video tracking of color-tagged insects. *eLife* **9**, e58145. (doi:10.7554/eLife.58145)

**Table S1.** Experimental design to test the effects of caregiver genotype on the growth and development of brood (Experiment 1). We controlled the age, number, and genotype of brood, and the age and number of caregivers. Thus, colonies differ primarily in the genotype of caregivers, permitting observation of how brood phenotypes are impacted by the rearing environment produced by caregivers of different genotypes. Related to Figures 1 and 2.

| Genotype of caregivers^1^ | A | B | D | M |
| --- | --- | --- | --- | --- |
| Genotype of supplied brood^2^ | **B** | **B** | **B** | **B** |
| # of experimental colonies | 8 | 8 | 8 | 8 |
| # of caregivers per colony | 25 | 25 | 25 | 25 |
| # of eggs supplied | 50 | 50 | 50 | 50 |
| Age of caregivers at brood transfer (days) | ~32 | ~35 | ~31 | ~32 |

^1^ Caregivers did not have vestigial eyes and were thus mostly regular workers.

^2^ All eggs were 10 ± 1 days old (i.e., a couple of days from larval hatching) and sourced from the same line B (STC6) stock colony.

**Table S2.** Experimental design of experiment 2, to (i) test the effect of caregiver genotype, age, and colony size on length of larval development, survival, and the propensity to develop as intercastes, (ii) test whether caregiver IGEs on brood phenotypes are conditional on other factors (caregiver age and colony size). ­We also tested if the body sizes of brood reared by line A caregivers are on average larger than those reared by B caregivers, at a colony size of 25 (see Figure S9). m.o. = month-old; d.o. = day-old. Related to Figure 3.

| Condition | Caregiver genotype (colony) | Brood genotype (colony) | Caregiver age | Colony size (workers) | Input (supplied with) | # of replicate colonies | Figure label |
| --- | --- | --- | --- | --- | --- | --- | --- |
| old A25 | A (C16) | B (STC6) | 1 m.o. | 25 | 50 1^st^ instar larvae | 5 | A_25 |
| old B25 | B (STC6) | B (STC6) | 1 m.o. | 25 | 50 1^st^ instar larvae | 5 | B_25 |
| old A50 | A (C16) | B (STC6) | 1 m.o. | 50 | 50 1^st^ instar larvae | 5 | A_50 |
| old B50 | B (STC6) | B (STC6) | 1 m.o. | 50 | 50 1^st^ instar larvae | 5 | B_50 |
| young A50 | A (C16) | B (STC6) | 5-7 d.o. | 50 | 50 1^st^ instar larvae | 5 | A_young |
| young B50 | B (STC6) | B (STC6) | 5-7 d.o. | 50 | 50 1^st^ instar larvae | 5 | B_young |

**Table S3**. Tukey’s multiple comparisons tests after repeated measures ANOVA for daily foraging activity between caregivers of different clonal lines, using the estimated average number of ants in the foraging chamber at any time as a proxy for foraging activity of colonies. Statistically significant differences (*p.adj* < 0.05) are in bold. In highlight are days in which line B caregivers were significantly more active than line A (turquoise) and line M (yellow) caregivers. Related to Experiment 1 and Figure S3*c*.

|  | **Tukey's multiple comparisons test adjusted *p* values (# of ants in arena)** | | | | | |  |
| --- | --- | --- | --- | --- | --- | --- | --- |
| **Day** | A vs. B | A vs. D | A vs. M | B vs. D | B vs. M | D vs. M |  |
| 1 | 0.8402 | **0.0316** | 0.6828 | **0.0105** | 0.0725 | **<0.0001** | 1st - 3rd instar |
| 2 | 0.9861 | 0.6249 | 0.9549 | 0.2994 | 0.5644 | 0.1013 |  |
| 3 | 0.9868 | 0.2753 | 0.1943 | 0.0721 | 0.0962 | **0.0018** |  |
| 4 | 0.9977 | **0.0294** | 0.3718 | **0.0495** | 0.614 | **0.0013** |  |
| 5 | 0.9276 | **0.0015** | 0.4878 | **0.004** | 0.1868 | **<0.0001** |  |
| 6 | 0.3427 | **0.0081** | 0.3304 | 0.1282 | **0.0169** | **0.0005** |  |
| 7 | 0.5209 | **0.0046** | 0.1154 | 0.4841 | **0.0344** | **0.0003** |  |
| 8 | **0.0108** | **0.0032** | 0.9997 | 0.6383 | **0.0059** | **0.0015** | 4th instar |
| 9 | **0.0029** | **0.0019** | 0.9863 | 0.9912 | **0.0001** | **<0.0001** |  |
| 10 | 0.3202 | 0.0553 | 0.9988 | 0.3072 | **0.0447** | **0.0011** |  |
| 11 | 0.0652 | **0.0002** | >0.9999 | **0.0206** | 0.0589 | **0.0002** |  |
| 12 | **0.0079** | **<0.0001** | 0.9345 | **0.0115** | 0.182 | **0.0018** |  |
| 13 | 0.0983 | **0.0001** | 0.4549 | **0.0092** | 0.9859 | **0.0368** |  |
| 14 | 0.09 | **<0.0001** | 0.5568 | **0.0134** | 0.9835 | 0.0512 |  |
| 15 | 0.1945 | **0.0006** | **0.0073** | **0.0281** | 0.9508 | **0.0256** |  |

**Table S4**. Tukey’s multiple comparisons tests after repeated measures ANOVA for daily foraging activity between caregivers of different clonal lines, using the estimated total distance travelled by ants in the foraging chamber as a proxy for foraging activity of a colony. Statistically significant differences (*p.adj* < 0.05) are in bold. In highlight are days in which line B caregivers were significantly more active than line A (turquoise) and line M (yellow) caregivers. Related to Experiment 1 and Figure 2*c*.

|  | **Tukey's multiple comparisons test adjusted *p* values (total distance travelled)** | | | | | |  |
| --- | --- | --- | --- | --- | --- | --- | --- |
| **Day** | A vs. B | A vs. D | A vs. M | B vs. D | B vs. M | D vs. M |  |
| 1 | 0.933 | 0.0546 | 0.6725 | **0.0119** | 0.0882 | **<0.0001** | 1st - 3rd instar |
| 2 | 0.9998 | 0.7024 | 0.7818 | 0.1382 | 0.4197 | **0.0276** |  |
| 3 | 0.9667 | 0.2777 | 0.1895 | 0.0608 | 0.1136 | **0.0024** |  |
| 4 | 0.9953 | **0.0263** | 0.2803 | **0.0391** | 0.5377 | **0.0003** |  |
| 5 | 0.94 | **0.0013** | 0.4968 | **0.0039** | 0.197 | **<0.0001** |  |
| 6 | 0.415 | **0.0069** | 0.2812 | 0.1139 | **0.0176** | **0.0004** |  |
| 7 | 0.3262 | **0.0019** | 0.1975 | 0.6617 | 0.0524 | **0.0002** |  |
| 8 | **0.0026** | **0.0005** | 0.9982 | 0.6892 | **0.002** | **0.0003** | 4th instar |
| 9 | **0.0011** | **0.0002** | 0.9983 | 0.9803 | **0.0006** | **<0.0001** |  |
| 10 | 0.1773 | **0.0225** | 0.9925 | 0.6083 | **0.0272** | **0.0002** |  |
| 11 | 0.0572 | **0.0001** | 0.9989 | **0.0338** | **0.0308** | **<0.0001** |  |
| 12 | **0.0054** | **<0.0001** | 0.9828 | **0.0269** | **0.0079** | **<0.0001** |  |
| 13 | 0.0575 | **<0.0001** | 0.8237 | **0.0104** | **0.011** | **<0.0001** |  |
| 14 | **0.0348** | **<0.0001** | 0.4519 | **0.0068** | 0.9271 | **0.0173** |  |
| 15 | 0.1938 | **0.0006** | **0.0345** | **0.0306** | >0.9999 | **0.0144** |  |

**Table S5**. Pairwise contrasts of estimated marginal means based on the linear model for length of larval development using the function *emmeans* in R. *p*-values were adjusted using the Benjamini & Hochberg method (for 28 tests) to control the false discovery rate. For colonies with 50 caregivers, there is a significant difference in length of larval development when comparing young and old B caregivers (**yellow**; direction = longer development with young caregivers), but no significant difference when comparing young and old A caregivers (**red**). This suggests an age effect specific to line B caregivers only. Also, when comparing old A caregivers at colony sizes of 25 and 50, larger colony size is associated with longer larval development (**green**), but not when comparing old B caregivers (**turquoise**). This suggests a colony size effect specific to line A caregivers only. There is a difference in length of larval development when comparing A and B caregivers at colony sizes of 50 (both old (**blue**) and young (**grey**)), but not at colony sizes of 25 (**purple**). This suggests a caregiver genotype effect on larval development conditional on larger colony size. Related to Experiment 2 and Figure 3*a*, 3*b*.

contrast estimate SE df t.ratio p.value

A old fifty - B old fifty -0.021842 0.004400 24 -4.964 0.0001

A old fifty - A young fifty -0.002830 0.005312 24 -0.533 0.6451

A old fifty - B young fifty -0.012336 0.004504 24 -2.739 0.0200

A old fifty - A old twentyfive -0.018676 0.004397 24 -4.247 0.0006

A old fifty - B old twentyfive -0.019544 0.004458 24 -4.384 0.0005

A old fifty - A young twentyfive -0.021506 0.009253 24 -2.324 0.0476

A old fifty - B young twentyfive -0.010038 0.004633 24 -2.167 0.0600

B old fifty - A young fifty 0.019012 0.003085 24 6.163 <.0001

B old fifty - B young fifty 0.009506 0.001260 24 7.547 <.0001

B old fifty - A old twentyfive 0.003166 0.000798 24 3.968 0.0011

B old fifty - B old twentyfive 0.002298 0.001085 24 2.118 0.0600

B old fifty - A young twentyfive 0.000336 0.005372 24 0.063 0.9507

B old fifty - B young twentyfive 0.011804 0.001851 24 6.377 <.0001

A young fifty - B young fifty -0.009506 0.003231 24 -2.942 0.0133

A young fifty - A old twentyfive -0.015846 0.003081 24 -5.144 0.0001

A young fifty - B old twentyfive -0.016714 0.003167 24 -5.277 0.0001

A young fifty - A young twentyfive -0.018676 0.004397 24 -4.247 0.0006

A young fifty - B young twentyfive -0.007208 0.003408 24 -2.115 0.0600

B young fifty - A old twentyfive -0.006340 0.001249 24 -5.075 0.0001

B young fifty - B old twentyfive -0.007208 0.001449 24 -4.973 0.0001

B young fifty - A young twentyfive -0.009170 0.005457 24 -1.681 0.1288

B young fifty - B young twentyfive 0.002298 0.001085 24 2.118 0.0600

A old twentyfive - B old twentyfive -0.000868 0.001073 24 -0.809 0.4974

A old twentyfive - A young twentyfive -0.002830 0.005312 24 -0.533 0.6451

A old twentyfive - B young twentyfive 0.008638 0.001654 24 5.221 0.0001

B old twentyfive - A young twentyfive -0.001962 0.005419 24 -0.362 0.7472

B old twentyfive - B young twentyfive 0.009506 0.001260 24 7.547 <.0001

A young twentyfive - B young twentyfive 0.011468 0.005564 24 2.061 0.0640

**Table S6**. Pairwise contrasts of estimated marginal means based on the linear model for brood survival using the function *emmeans* in R. Results are averaged over the levels of age. *p*-values were adjusted using the Benjamini & Hochberg method (for 6 tests) to control for multiple comparisons. Line A caregivers had better brood survival at colony sizes of 25 (**green**), but colony size had no effect for line B caregivers (**turquoise**). This suggests a colony size effect specific to line A. Line B caregivers outperformed line A caregivers at colony sizes of 50 (**grey**), but line A outperformed line B caregivers at colony sizes of 25 (**purple**). This suggests a caregiver IGE on brood survival that changed its direction in response to colony size. Related to Experiment 2 and Figure 3*c*.

contrast estimate SE df t.ratio p.value

A fifty - B fifty -16.2 3.79 24 -4.276 0.0009

A fifty - A twentyfive -22.6 5.36 24 -4.218 0.0009

A fifty - B twentyfive -5.8 5.36 24 -1.082 0.2898

B fifty - A twentyfive -6.4 5.36 24 -1.194 0.2898

B fifty - B twentyfive 10.4 5.36 24 1.941 0.0962

A twentyfive - B twentyfive 16.8 6.56 24 2.560 0.0344

**Table S7**. Pairwise contrasts of estimated marginal means based on the generalized linear mixed model for proportion of intercaste brood using the function *emmeans* in R. *p*-values were adjusted using the Benjamini & Hochberg method (for 28 tests) to control the false discovery rate. For colonies with 50 old caregivers, there is a significant difference in the proportion of intercaste brood reared when comparing A and B caregivers (**yellow**; direction = more intercastes with A caregivers). Also, when comparing old caregivers of line A and B at colony sizes of 25, line A caregivers reared a higher proportion of intercaste brood (**green**). Lastly, for colonies with 50 young caregivers, line A caregivers reared a higher proportion of intercaste brood compared to line B caregivers (**red**). Thus, the caregiver genotype effect on brood intercaste proportions was consistent, and not conditional on context. Related to Experiment 2 and Figure 3*d*.

contrast odds.ratio SE df null z.ratio p.value

A old fifty / B old fifty 4.156 2.4373 Inf 1 2.429 0.0424

A old fifty / A young fifty 1.396 0.8111 Inf 1 0.574 0.6093

A old fifty / B young fifty 11.428 7.4937 Inf 1 3.715 0.0040

A old fifty / A old twentyfive 1.753 0.9829 Inf 1 1.001 0.4035

A old fifty / B old twentyfive 10.016 7.1115 Inf 1 3.245 0.0055

A old fifty / A young twentyfive 2.447 2.5239 Inf 1 0.867 0.4696

A old fifty / B young twentyfive 27.542 25.6125 Inf 1 3.566 0.0040

B old fifty / A young fifty 0.336 0.1745 Inf 1 -2.100 0.0833

B old fifty / B young fifty 2.750 1.6558 Inf 1 1.680 0.1532

B old fifty / A old twentyfive 0.422 0.2081 Inf 1 -1.750 0.1497

B old fifty / B old twentyfive 2.410 1.5897 Inf 1 1.334 0.2552

B old fifty / A young twentyfive 0.589 0.4489 Inf 1 -0.695 0.5684

B old fifty / B young twentyfive 6.627 6.8628 Inf 1 1.826 0.1356

A young fifty / B young fifty 8.186 4.8896 Inf 1 3.520 0.0040

A young fifty / A old twentyfive 1.255 0.6150 Inf 1 0.464 0.6662

A young fifty / B old twentyfive 7.175 4.7065 Inf 1 3.004 0.0093

A young fifty / A young twentyfive 1.753 0.9829 Inf 1 1.001 0.4035

A young fifty / B young twentyfive 19.729 17.5576 Inf 1 3.351 0.0055

B young fifty / A old twentyfive 0.153 0.0885 Inf 1 -3.249 0.0055

B young fifty / B old twentyfive 0.876 0.6339 Inf 1 -0.182 0.8554

B young fifty / A young twentyfive 0.214 0.1755 Inf 1 -1.880 0.1293

B young fifty / B young twentyfive 2.410 1.5897 Inf 1 1.334 0.2552

A old twentyfive / B old twentyfive 5.715 3.6376 Inf 1 2.739 0.0192

A old twentyfive / A young twentyfive 1.396 0.8111 Inf 1 0.574 0.6093

A old twentyfive / B young twentyfive 15.715 13.7827 Inf 1 3.141 0.0067

B old twentyfive / A young twentyfive 0.244 0.2106 Inf 1 -1.635 0.1588

B old twentyfive / B young twentyfive 2.750 1.6558 Inf 1 1.680 0.1532

A young twentyfive / B young 25 11.257 11.8515 Inf 1 2.299 0.0547


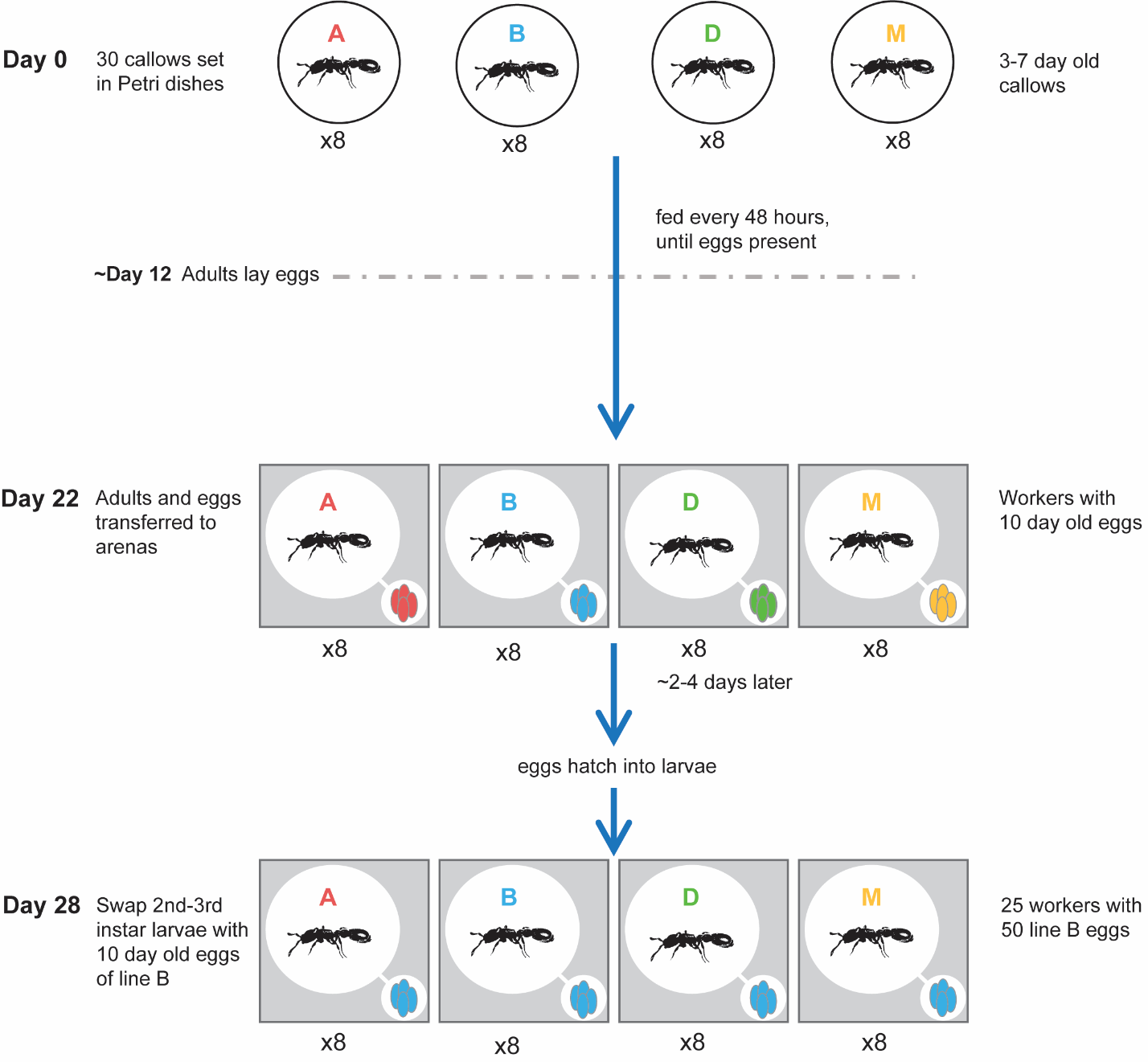


**Figure S1.** Visual illustration of experimental design and setup for Experiment 1 (related to Table S1). For each line, eight colonies comprised of 30 regular callow workers (3-7 days old) were set in 50mm diameter Petri dishes with a plaster of Paris floor. Each colony was fed fire ant brood every 48 hours until eggs were present. Each colony was transferred to a raiding arena 22 days later, at which point colonies had eggs that were approximately 10 days old. A brood swap was done on day 28, when all colonies had 2^nd^-3^rd^ instar larvae – larvae were replaced with 50 line B eggs that were approximately 10 days old. On the day we performed the brood swap, we adjusted all colonies to have exactly 25 regular workers. Related to Methods and Materials.

**
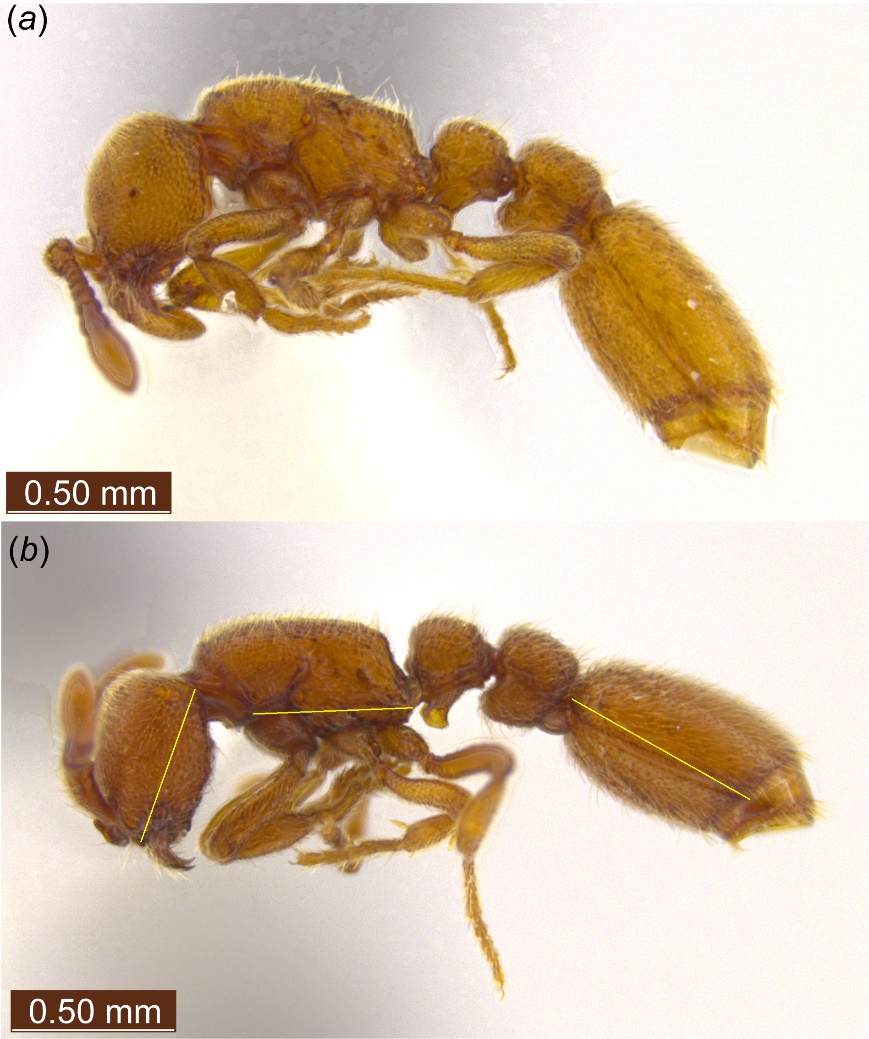
­**

**Figure S2.** Distinguishing regular workers and intercastes by the presence of vestigial eyes, and illustration of how body size was measured in this study. (*a*) Image of an intercaste, with arrow pointing at small vestigial eye spot. (*b*) Image of a regular worker without vestigial eye spot. To estimate body size, we measured and summed the length of the head, thorax, and 1^st^ gastral segment from a lateral view (shown by yellow lines). We used landmarks on the head, thorax, and abdomen for consistent measurements across ants. We only include the first gastral segment because the abdomen is telescoping. Related to Methods and Materials.

**
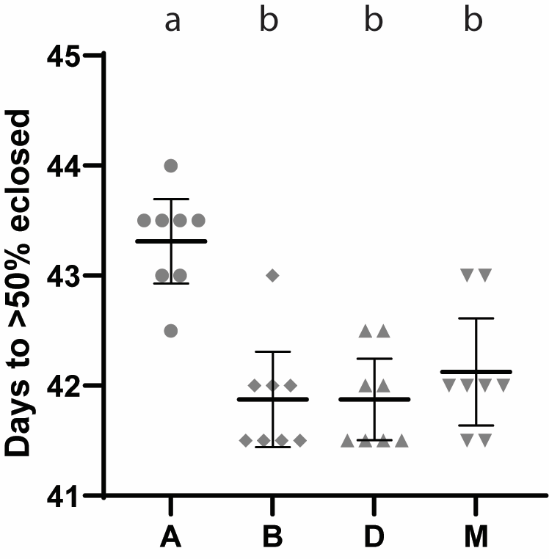
**

**Figure S3.** Caregiver genotype influenced the time to eclosion of brood (one-way ANOVA, *p* < 0.0001). Brood reared by line A caregivers reached adulthood later compared to all other lines (Tukey’s HSD: A vs. B, *p* < 0.0001; A vs. D, *p* < 0.0001; A vs. M, *p* = 0.0003). Caregiver genotypes are labelled on the x-axis. Each point represents a single colony; bars represent 95% C.Is of the mean. Related to Experiment 1.

**
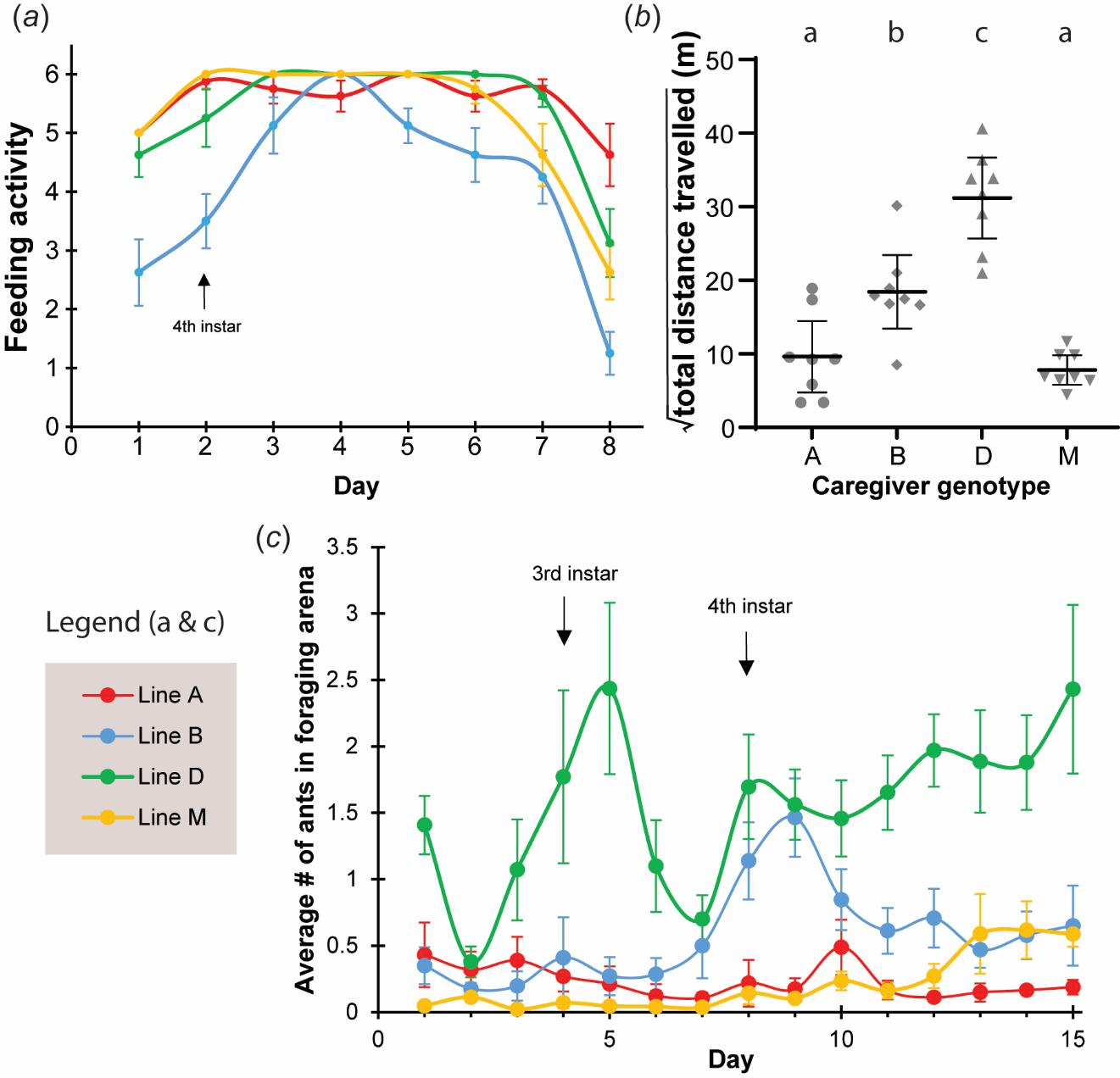
**

**Figure S4.** Feeding and foraging activity of colonies that vary by caregiver genotype. (*a*) Daily feeding scores for four caregiver genotypes, showing that colonies with line B caregivers consumed less food on average compared to those with line A, D and M caregivers on many days. On day four, about two days after larvae become 4^th^ instars, line B colonies had peak feeding activity. Day one corresponds to late 3^rd^ instar larvae (day 7 in S3*c*). Bars represent SEM. (*b*) When comparing the total distance travelled by ants in the foraging chamber during the entire brood care phase, the clonal lines significantly differed in their cumulative foraging activity. Caregivers of line D were most active (Tukey’s HSD: A vs. D, *p* < 0.0001; B vs. D, *p* = 0.0004; D vs. M, *p* < 0.0001), followed by line B (Tukey’s HSD: A vs. B, *p* = 0.0156; B vs. M, *p* = 0.0030), and then lines A and M (i.e., D>B>A=M); bars represent 95% C.Is of the mean. (*c*) Daily foraging activity of colonies (estimated by quantifying the average number of ants in the foraging arena at any given time for that day) that vary only in caregiver genotype. Line D caregivers were more active than line A and M caregivers on most days, while line B caregivers were more active than line A and M on days 8 and 9 when larvae became 4^th^ instars (see Table S3). Bars represent SEM. Related to Experiment 1.


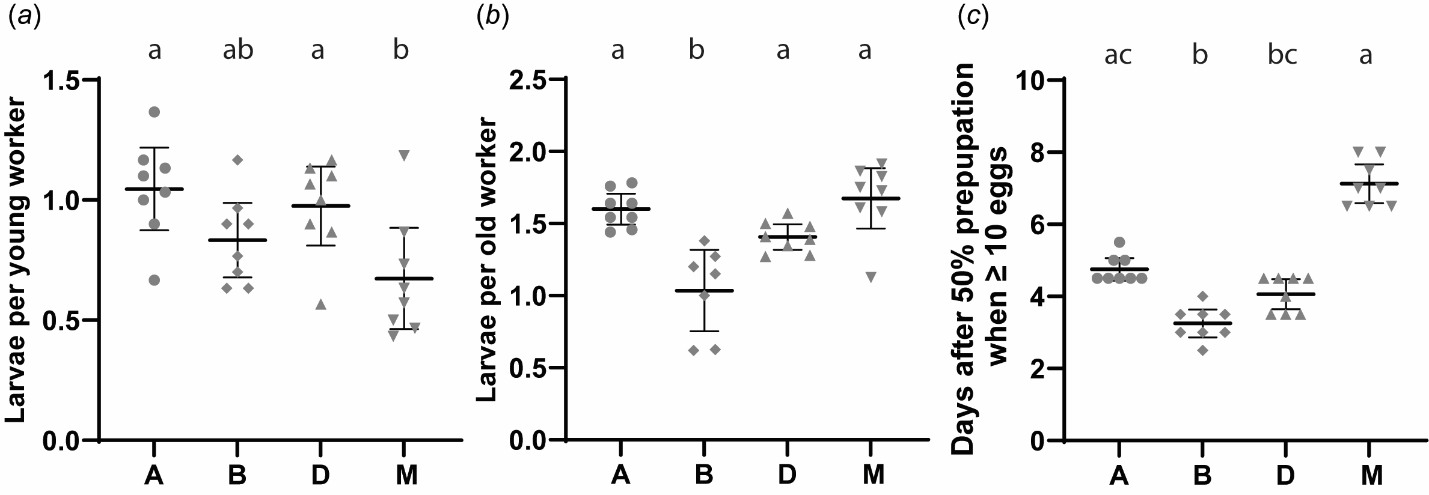


**Figure S5.** Differences in reproductive traits between the four clonal lines. (*a*) Looking at the first cohort of larvae produced by the young (16-20 days old) caregivers, lines A and D produced significantly more larvae than line M (Tukey’s HSD: A vs. M, *p* = 0.0076; D vs. M, *p* = 0.0381). (*b*) When comparing workers that are 51-55 days old, line A, D and M workers produced more larvae per worker than line B adults (Tukey’s HSD: A vs. B, *p* = 0.0001; B vs. D, p = 0.0103; B vs. M, *p* < 0.0001). (*c*) Genotype influenced the number of days until at least 10 eggs were laid after at least 50% of brood were prepupae (Kruskal-Wallis test, *p* < 0.0001). Line M took significantly longer to lay eggs after the end of the brood care phase compared to lines B and D, but not A (Dunn’s multiple comparisons test: B vs. M, *p* < 0.0001; D vs. M, *p* = 0.0039; A vs. M, *p* = 0.2711). Also, line A took significantly longer to lay eggs after the brood care phase compared to line B, but not D and M (Dunn’s multiple comparisons test: A vs. B, *p* = 0.0174; A vs. D, *p* = 0.9557; A vs. M, *p* = 0.2771). Caregiver genotypes shown on x-axis; Bars represent 95% C.Is of the mean. Related to Experiment 1.


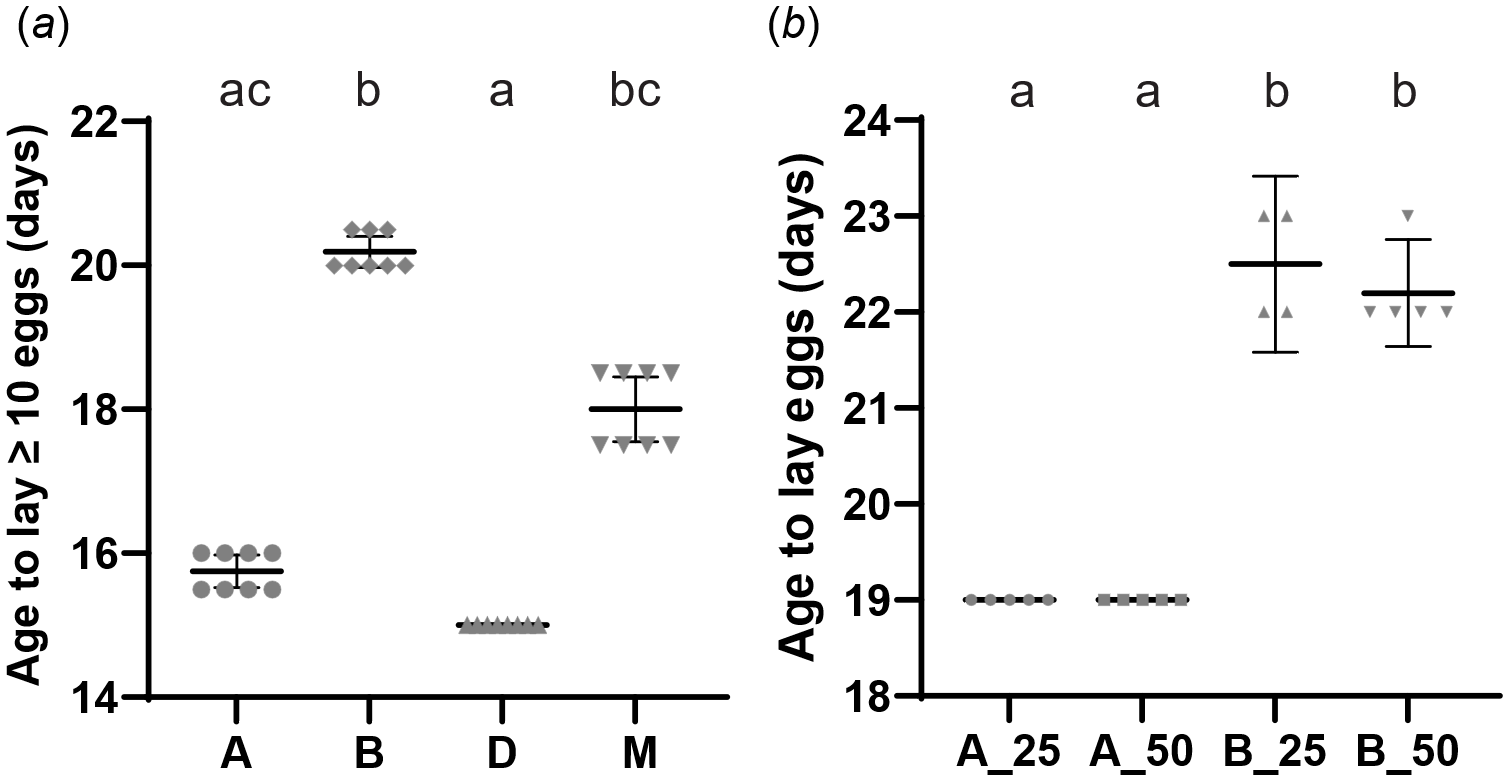


**Figure S6.** Reproductive maturation of four clonal lines. (*a*) As part of the first experiment, eight colonies per clonal line comprised of 30 regular workers were set and the date when at least 10 eggs were laid by the workers was recorded. The age of workers when at least 10 eggs were present differed between genotypes (Kruskal-Wallis test, *p* < 0.0001). Line A and D workers laid eggs at a younger age than line B workers, and line D workers laid eggs at a younger age than line M workers (Dunn’s multiple comparisons test: A vs. B, *p* = 0.0033; B vs. D, *p* < 0.0001; D vs. M, *p* = 0.0033). All colonies regardless of clonal line were set on the same day, but workers of different clonal lines differed in age on that day (A = ~4 days old; B = ~7 days old; D = ~3 days old; M = ~4 days old). Related to Experiment 1. (*b*) As part of Experiment 2, colonies of either 25 or 50 regular workers for both line A and B were set up and the date when at least 10 eggs (in the case of colonies with 25 workers) or 20 eggs (in the case of colonies with 50 workers) were present was recorded. In this experiment, the line A and B workers used were the same age. Line A workers laid eggs at a younger age than line B workers. Related to Experiment 2. Together, (*a*) and (*b*) suggest that reproductive maturation in line A workers is faster than in line B workers.


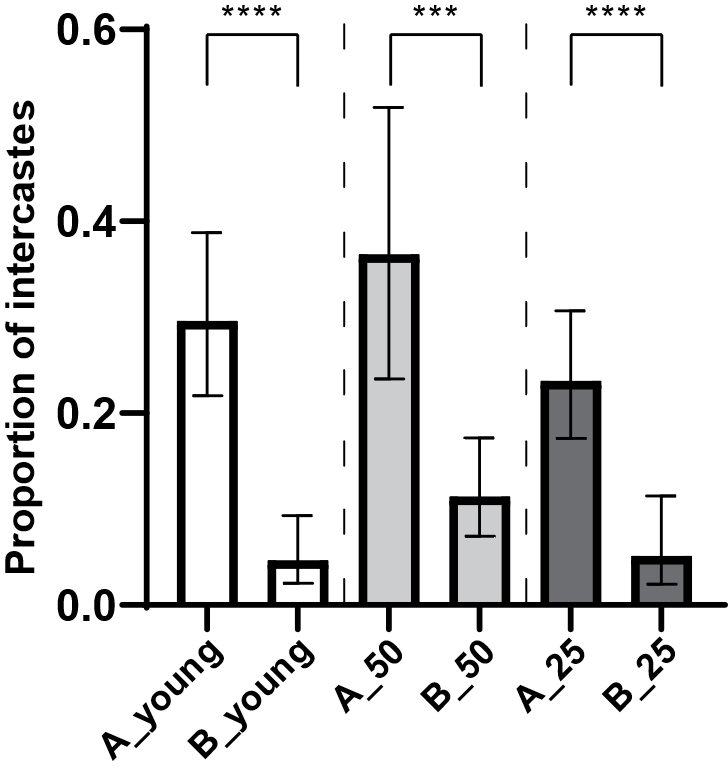


**Figure S7.** Effect of caregiver genotype on the proportion of intercaste brood. Proportion of intercaste brood reared in different conditions, where each shade denotes a controlled comparison between line A and B caregivers. Conditions are labelled on the x-axis (described in the methods and table S2). All three comparisons between A and B caregivers show that a higher proportion of brood developed into intercastes when reared by line A caregivers (Fisher’s exact tests: A_young vs. B_young, *p* < 0.0001; A_50 vs. B_50, *p* < 0.001; A_25 vs. B_25, *p* < 0.0001). Bars represent 95% C.Is of the mean. Related to Experiment 2 and Figure 3*d*.


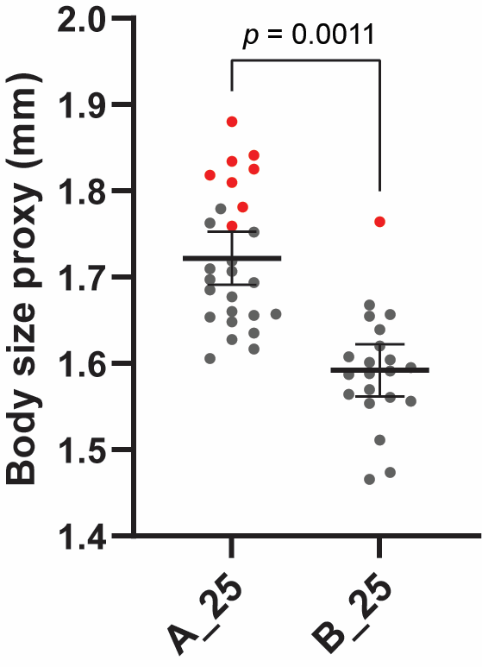


**Figure S8.** A linear mixed model, with caregiver genotype as the fixed effect and colony as a random effect, showed that caregiver genotype influenced the body size of brood (Type II ANOVA of LMM: χ²(1) = 27.99, *p* = 1.22^-07^). Brood reared by line A caregivers were on average larger than those reared by line B caregivers at colony sizes of 25 (A vs. B: *t* = 5.284, *p* = 0.0011), even when excluding intercastes from the analysis (*t* = 4.884, *p* = 0.002). Red and grey dots indicate intercaste and regular worker brood, respectively. Conditions on x-axis are listed in Table S2. Bars represent 95% C.Is of the mean. Related to Experiment 2.


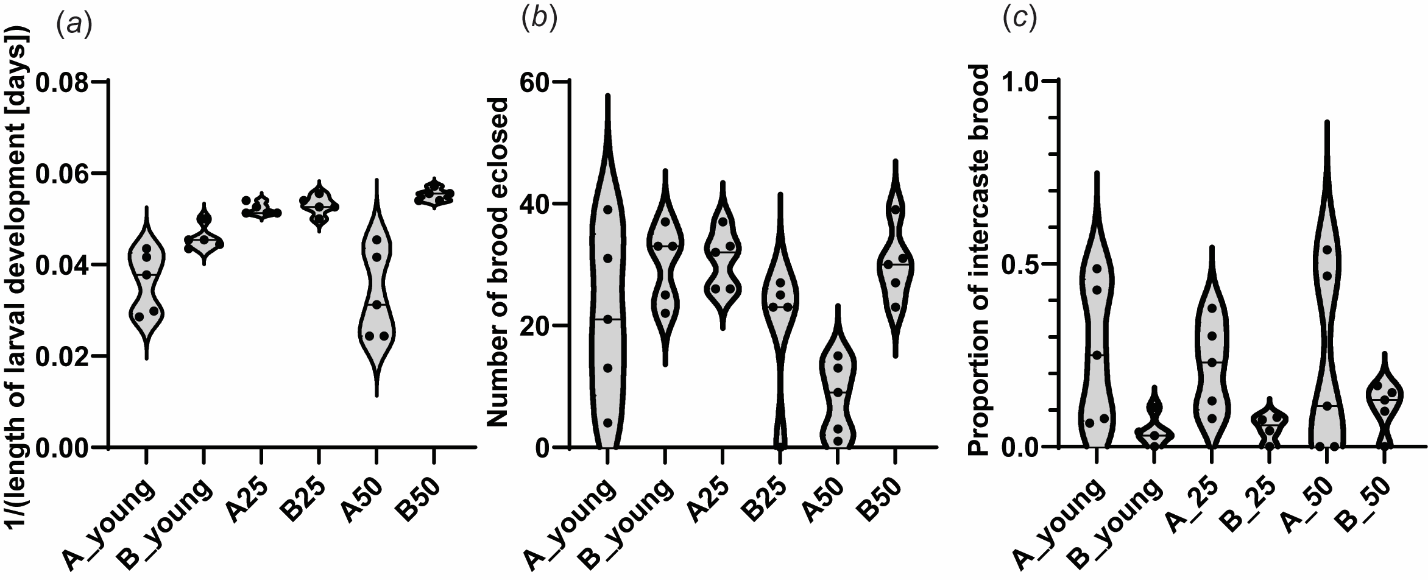


**Figure S9.** Violin plots of (*a*) length of larval development in days (reciprocal transformed), (*b*) number of brood surviving to adult eclosion, and (*c*) proportion of intercastes reared across conditions that vary in caregiver age and number (described in Table S2). Related to Experiment 2, Figure 3*a-c*, and Tables S5, S6.
